# PEDOT/Polypyrrole Core–Sheath Fibers for Use as Conducting Polymer Artificial Muscles

**DOI:** 10.1021/acsami.4c17667

**Published:** 2025-01-16

**Authors:** Mathis Bruns, Shayan Mehraeen, Jose G. Martinez, Chokri Cherif, Edwin W. H. Jager

**Affiliations:** † Institute of Textile Machinery and High Performance Material Technology (ITM), TUD Dresden University of Technology, 01062 Dresden, Germany; ‡ Sensor and Actuator Systems, Department of Physics, Chemistry and Biology (IFM), Linköping University, Linköping SE-581 83, Sweden

**Keywords:** actuators, yarns, smart textiles, wearables, iEAPs, biomimetic

## Abstract

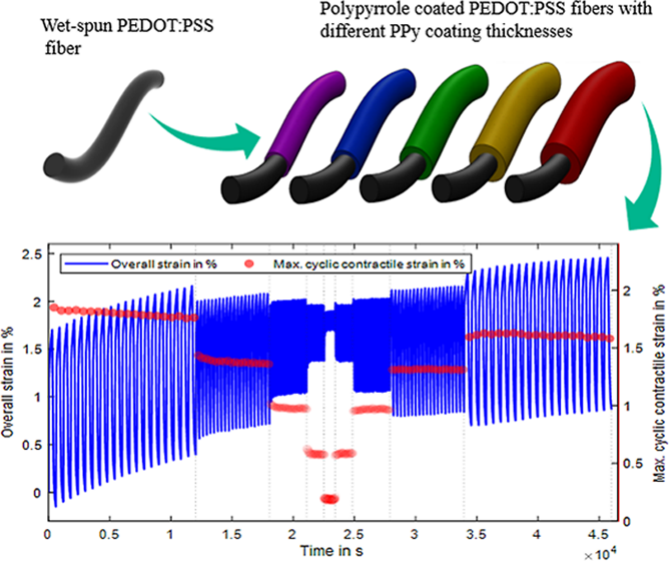

Electropolymerized polypyrrole (PPy) is considered as one of the promising polymers for use in ionic-electroactive or conducting polymer (CP) actuators. Its electromechanical properties surpass those of other prominent CPs such as poly(3,4-ethylenedioxythiophene) polystyrene sulfonate (PEDOT/PSS) or polyaniline. However, freestanding and linear contracting actuator fibers made solely of electropolymerized PPy are not available yet. This work therefore targets the development of all-CP-based actuator fibers: electromechanically active PPy is electropolymerized on the surface of wet-spun, also electromechanically active PEDOT/PSS fibers. The thickness of the PPy fiber sheath is varied by using different electropolymerization durations. Mechanical and actuation properties of the different PEDOT/PPy core–sheath actuator fibers are investigated via tensile tests and isotonic actuation strain and isometric actuation force measurements, respectively. The fiber actuators show high tensile stability in both dry and aqueous conditions, rendering them highly suitable for actuation in aqueous electrolyte media. Regarding linear, untwisted, and uncoiled CP fiber actuators, the presented actuation measurements demonstrate to the best of our knowledge the highest reported linear contractile actuation strains of up to 2.2% in electrolytes and remarkable tensile actuation stresses of 1.64 MPa, as well as a high long-term cyclic actuation stability using varying actuation durations. This renders the fibers as a highly promising material, particularly with regard to their further structural textile processing for use in actuating wearables or soft robots.

## 1 Introduction

Soft robotics is a growing research area that offers the potential to complement conventional robot structures with active, flexible, and stretchable materials. This creates a basis for a multitude of new interactive application possibilities, including direct human–machine interaction.^1^

Besides soft robotics, mechanically active wearables are gaining a growing interest in both research and industry.^2^ These sensor- or actuator-equipped garments are capable of providing information on the medical or physical conditions of the human body, including temperature, moisture, and sweat^3^ and can actively control the permeability of the garment itself in response to the sensed parameters. They can also sense pressure and lead to compression^4,5^ or sense and actuate in terms of textile-based exoskeletons.^6,7^ For such applications, the integration of sensor and actuator functions into the textile, and even directly into the fiber, can lead to robust applications with a multitude of the before mentioned sensor and actuator functions.^8,9^ Recent advancements in this field have notably focused on improving the performance and integration of actuating fibers. For instance, thermally activated fiber-shaped actuators, such as shape memory alloys or semicrystalline fibers with orientation changes that result in actuation, have been integrated in textiles and soft robotics.^10,11^ Moreover, actuators driven by electric fields, such as dielectric elastomer actuators^12^ or fluid pressure-driven actuators,^13^ have been adapted for integration into textiles. While these textile or fiber actuators have demonstrated excellent performance in specific actuation metrics, such as actuation strain, bandwidth, strength, power density, or efficiency, they still face the challenge of gaining high performances in all these metrics at the same time.^14^

Conducting or conjugated polymers (CPs) offer great potential for fiber-based sensors and actuators based on their unique properties: first, these materials combine the benefits of polymeric materials such as flexibility for a simple textile processing and lightweight for energy-efficient movement of such wearables or components with outstanding electrical performance, comparable to semiconductors or metals which are attributed due to their conjugated polymeric backbone and delocalization of π-electrons.^15,16^ Second, their usage as gas sensors or biosensors with specific sensitivities or as flexible strain sensors underscores their utility for the discussed applications.^17−20^ Additionally, CP is a representative of volume change materials,^21^ where volume change is driven by reversible reduction and oxidation of the polymer that requires only a low actuation voltage of 0.1–3.0 V. If an electrolyte with appropriate cations and anions is available, reduction and oxidation cause migration of cations or anions and solvent molecules into or out of the polymeric matrix to maintain charge equilibrium.^22−25^ The resulting change in volume can be exploited as mechanical work for use as actuators or artificial muscles.^14,23,26^

The most prominent CPs used in soft actuators are polyaniline,^27−29^ poly(3,4-ethylenedioxythiophene) (PEDOT),^30,31^ and especially polypyrrole (PPy).^32−34^

To highlight the characteristic properties of the polymers in terms of usage as actuators, PEDOT, in combination with the counterion polystyrene sulfonate (PSS), is water soluble (and therefore easy to process) and offers high conductivity,^35^ however offers only small actuation unless it is structurally processed such as folded or coiled. PPy is hardly soluble and challenging to process but offers the highest actuation performances reported. Electrochemical strains for PPy actuators are typically in the range of 2–5% for small strips,^26^ although volume changes of 30–40% have been reported for perpendicular expansion^36^ or for one excitation cycle for a PPy-based thin-film actuator.^37^

In order to realize fiber-shaped CP actuators, commercial yarns were dip-coated in PEDOT, followed by electrodeposition of PPy on the yarn.^32^ Optimization of the core yarn resulted in over an 11-fold increase in linear strain, highlighting the substantial influence of the passive core yarn on actuator performance.^33^ However, the passive core yarn still impedes efficiency, consuming much of the generated energy and adding dead weight. The use of all-CP yarns would eliminate these drawbacks. Additionally, for practical integration into fabrics via knitting or weaving, continuous fabrication of such yarns is essential as tens of meters are typically required per garment.

Besides this, PEDOT and PPy have been wet-spun in previous studies.^38−42^ Pristine PPy fibers were wet-spun using highly hazardous dichloroacetic acid as a solvent in the spinning dope, leading to fibers’ tensile strength of 25 MPa, 2% elongation at break, and an electrical conductivity of 566.8 S cm^–1^.^39,43^ These fibers were also investigated regarding their actuation performance, whereas the actuation remained under 1% linear strain, but it is worth to note that PPy fibers were synthesized by emulsion polymerization, whereas it is widely accepted that electropolymerized PPy exhibits the highest electromechanical performance.^44^ Wet-spun PEDOT-based fibers surpass wet-spun PPy-based fibers in terms of spinnability and mechanical properties, reaching higher tensile strengths of up to 956 MPa and electrical conductivities of more than 4000 S cm^–1^.^40,41^

In our last study, we investigated the electromechanical properties of wet-spun PEDOT/PSS fibers and found repeatable linear contractions of up to 0.56% of the straight fibers.^42^ Another study found that coiling a PEDOT fiber bundle increased the contractile strain induced by volume change by up to 11%,^45^ but such coiled yarns are difficult to integrate in a fabric using continuous production such as knitting and weaving.

In contrast, the integration of straight, linear actuator yarns into textile structures like knits and weaves and its effect on linear actuation properties was examinated, demonstrating a remarkable 53-fold increase in actuation strain when a single actuator yarn was incorporated into a knit structure.^32^ This demonstrates the high potential for linear actuating fibers for active textiles even if their initial single yarn linear strain is relatively low.

In addition to the resulting assessment that textile processing can significantly improve the actuation properties of CP fiber-based actuators, the question arises as to how a straight textile-processable actuator fiber, devoid of any passive core fibers that might impede actuator functionality, can amplify the performance of CP fiber actuators and enhance their suitability, particularly in view of soft robots and wearables. To address this, we present herein the manufacture of straight linear PEDOT/PPy core–sheath actuator fibers with varied PPy sheath thicknesses. The mechanical and actuation properties of the different fiber actuators are investigated and are presented.

## 2 Results and Discussion

**Figure 1:**
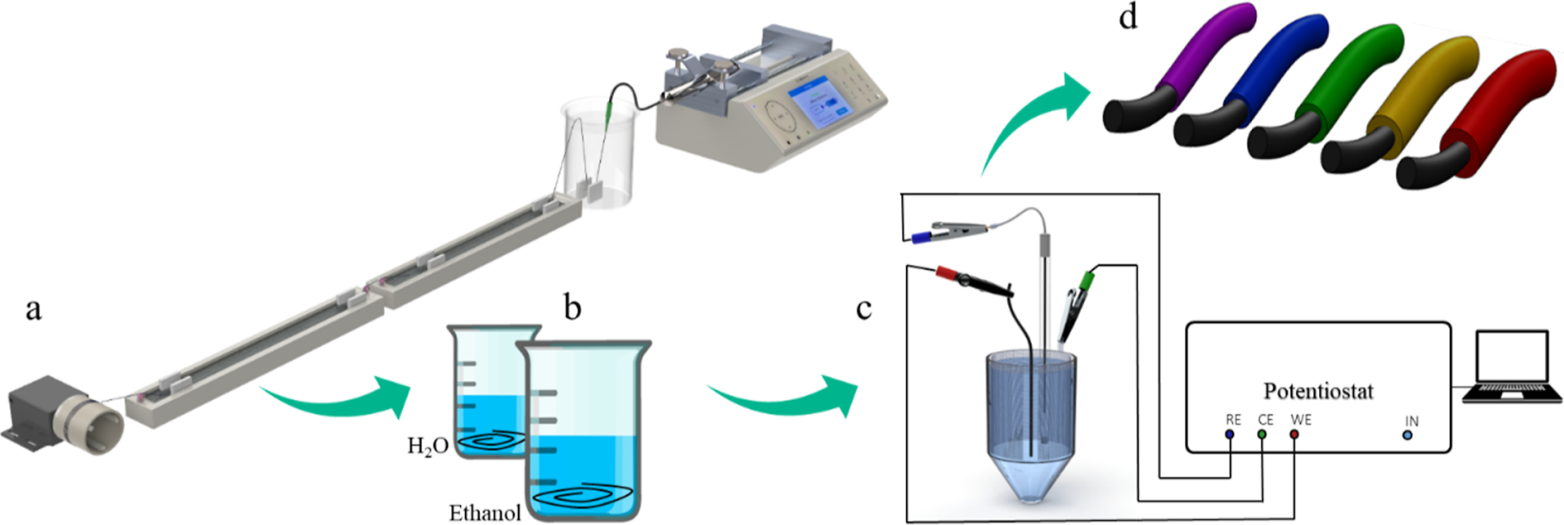
Production process of the PEDOT/PPy core–sheath fiber actuators. Wet-spinning of the PEDOT/PSS fibers (a), washing step of the obtained fibers (b), setup for electrochemical deposition of PPy onto the PEDOT/PSS fiber surface (c), and PEDOT/PPy core–sheath actuator fibers with different PPy layer thicknesses (d). (a) Reproduced from ref (42). Available under a CC-BY 4.0 license © 2024 The Authors. Advanced Intelligent Systems published by Wiley-VCH GmbH.

In order to realize exclusively CP-based, highly electroactive PEDOT/PPy core–sheath fibers, we used wet-spun PEDOT fibers and electropolymerized PPy onto the fibers. The continuous wet-spinning process of the PEDOT/PSS fibers and their mechanical and electrochemical actuation properties are reported in our previous work in detail.^42^ Briefly, a PEDOT/PSS spinning solution with a solid content of 2.5 and 5 wt % dimethyl sulfoxide (with respect to the total solution mass) was extruded in a coagulation bath containing 20 wt % sulfuric acid (96%) in isopropanol. Afterward, the fiber was rapidly dried using an acetone bath to prevent them from sticking to each other during winding on the bobbin (Figure 1a). After they were wet-spun, the fibers were washed using deionized water and ethanol to quickly dry them (Figure 1b). Table 1 shows the properties of PEDOT fibers used for the subsequent electrodeposition of PPy onto the fiber surface to realize highly electroactive PEDOT/PPy core–sheath fibers.

**Table 1:** Properties of the Used PEDOT Fiber Actuator^a^

tensile strength	conductivity	max. actuation stress	max. linear contractile actuation strain
135 ± 8 MPa	565.2 ± 15.5 S cm^–1^	1.35 MPa	0.56%

a Data from Bruns et al.^42^

It is worth noting that mechanical strain had little effect on the conductivity of the fibers. All PPy fiber sheaths were realized by galvanostatic electrochemical deposition of PPy onto the PEDOT fiber surface as follows: a PEDOT fiber, which served as the working electrode, was immersed in an electrochemical cell containing a 0.1 M pyrrole monomer in 0.1 M aq. NaDBS solution (Figure 1c). To realize PEDOT/PPy core–sheath fibers with different PPy sheath thicknesses (Figure 1d), a constant current of 0.5 mA was set during different time periods, namely, 2500, 5000, 7500, 10,000, and 12,500 s.

We used DBS^–^ as the dopant since DBS^–^ anions are relatively large and therefore immobile in the PPy polymer network, as is the PSS in the PEDOT.^46−48^ Hence, the actuation using the NaDBS electrolyte is cation-driven for both polymers, leading to a smooth contraction of both actuator fiber components (PEDOT fiber core and PPy fiber sheath) at oxidation since Na^+^ cations and H_2_O molecules migrate out of the polymer networks to maintain charge equilibrium and vice versa when the polymers are reduced.^33,42^

### 2.1 Polypyrrole Electrochemical Deposition

**Figure 2:**
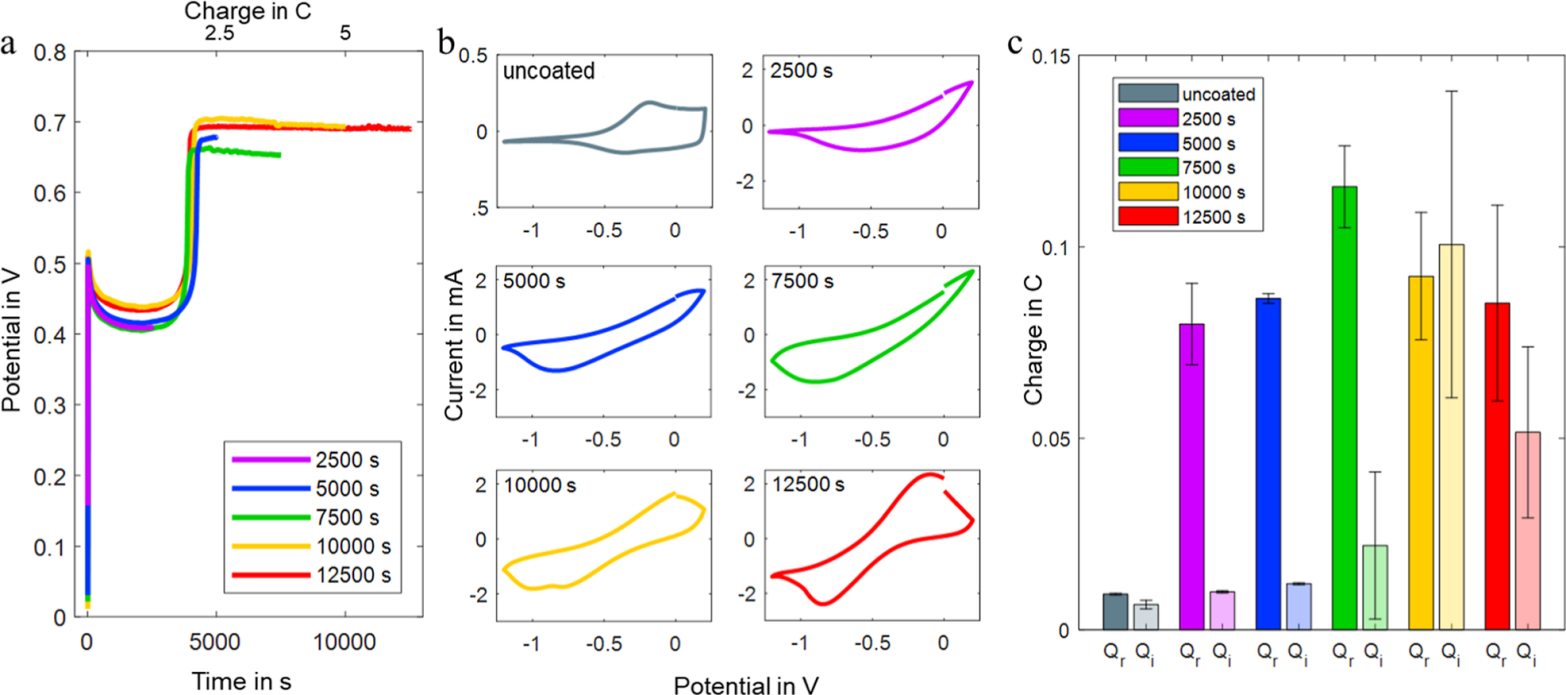
Electrochemical analysis of the electrodeposition of PPy on the PEDOT fiber surface. Potential over time or consumed charge during a constant applied current of 0.5 mA (a), third cycle of cyclic voltammograms before and after electropolymerization of different durations showing increasing potential peaks over increasing electrodeposition durations (b), and reversible (Q_r_) and irreversible (*Q*_i_) charge consumed during the cyclic voltammetry (CVs) of (b) (c).

Figure 2a shows the potential over time that was recorded during the electropolymerization. As previously reported,^23,33^ a capacitive double-layer charging and nucleation process was represented in an initial peak after the start of electrodeposition, followed by the growth process of the doped PPy and simultaneous oxidation of the PEDOT core fiber, represented by a plateau after the nucleation peak. Following this, an increase in the potential was recorded between 1.8 and 2.2 C (3600–4400 s polymerization duration), followed by a second plateau. The increase could be attributed to the outer polymeric network layer of the PEDOT fibers being filled with PPy,^49,50^ the fiber being fully covered by PPy.^49^ We assume that small standard deviations in the PEDOT core fiber’s circumferential area and electrical resistance^42^ are responsible for the small inequalities in potential evolutions over time for the different polymerization experiments.

We assessed the oxidation and reduction characteristics of the PEDOT fiber directly before PPy electrodeposition and afterward using CV between −1.2 and 0.2 V at a scan rate of 10 mVs^–1^. Figure 2b presents the third cycle for the uncoated PEDOT core fiber and the different PEDOT/PPy core–sheath fibers. With higher anodic and cathodic currents at the PEDOT/PPy core–sheath fibers compared to the uncoated PEDOT fiber, the measurements indicated PPy deposition. Looking at the CVs, it is also possible to observe that fibers with electrodeposition durations below 10,000 s showed increased currents as compared to the uncoated PEDOT fiber, indicating PPy synthesis on the PEDOT. This is confirmed by the higher reversible charges consumed during the CVs (Figure 2c), which increased with increasing electrodeposition times. However, they did not reveal clear characteristic oxidation peaks with the potential range and scan rate used for the experiment, while those fibers polymerized with a duration of 10,000 s and more showed clear characteristic oxidation and reduction peaks of PPy, pointing to a conformal PPy electrodeposition. Looking at the consumed charge for these samples, it was possible to observe that it was not as reversible as with thinner coatings, pointing to the existence of parallel irreversible reactions.^51^

### 2.2 Physical Properties of PEDOT/PPy Fibers

**Figure 3:**
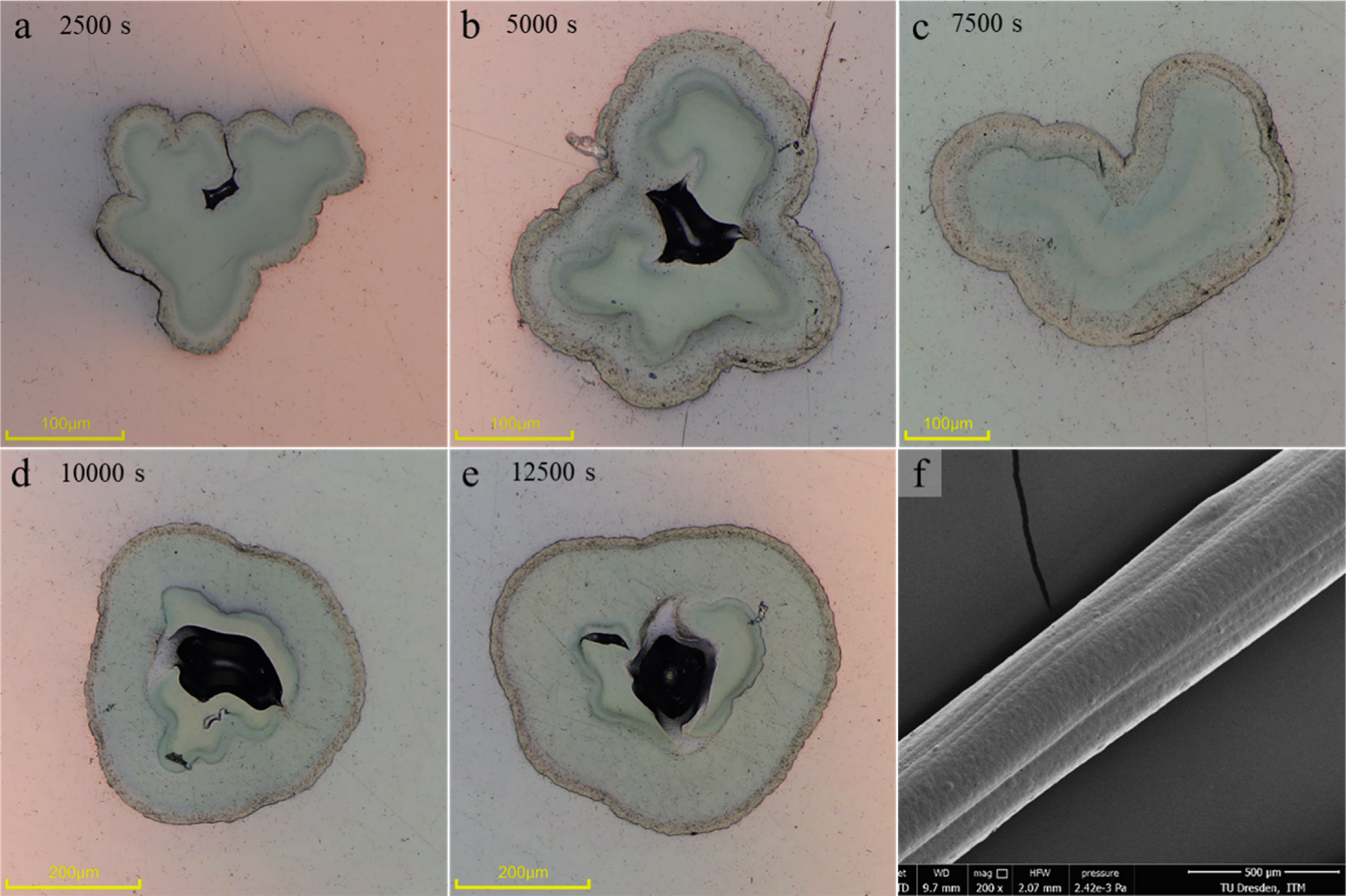
Cross-sectional images with different electropolymerization durations and side view on the PEDOT/PPy fibers in the dry state with optical images of a pure PEDOT core and an outer PEDOT/PPY layer for electrodeposition durations of 2500 s (a), 5000 s (b), 7500 s (c), 10,000 s (d), and 12,500 s (e). SEM side view on a PEDOT/PPy fiber with 5000 s PPy electrodeposition duration (f). Note the different scale bars on the images.

To determine the cross-sectional distribution of PPy on the PEDOT core fiber, the fibers were prepared as microsectioned specimens using epoxy resin. Figure 3a–e presents images of the dry PEDOT/PPS core–sheath fiber cross-sectional areas with all examined electrodeposition durations, captured with a digital light microscope. Figure 3f displays a scanning electron microscope-captured side view of such a fiber.

The images reveal that the PEDOT core fibers had no circular cross-section which is attributed to the rapid coagulation process while wet-spinning, leading to a collapse of the initial round fiber cross-section directly after the spinneret.^42^ Consequently, small hollow areas became entrapped by the growing PPy layer, visible as a darker area in Figure 3a,b,d,e. The vacuum used to prepare the microsectioned specimens could make the PEDOT core fiber crack open even more at the hollow areas.

In addition, no clear separation between PEDOT and PPy layers could be observed, but at least three different diffused layers. It could be hypothesized that those three layers could be an outer PPy layer, an intermediate layer composed of PPy and PEDOT, and an inner PEDOT layer.

This underlaying PEDOT core fiber cannot be considered only as a conductive layer, but PEDOT itself can also be oxidized and reduced.^52^ Thus, during polymerization, both processes are expected to occur: the oxidation of the PEDOT and the electropolymerization of PPy. Thus, as we did there, selecting the appropriate electropolymerization conditions is key to avoid overoxidation (degradation) of the underlaying PEDOT core fiber and, thus, maintain its conductivity and the mobility of the PEDOT chains. At the same time, the proper conditions for a good polymerization of PPy need to be considered. In this way, it should be possible to obtain good ionic and electrical conductivity between the PEDOT and PPy layers, contributing to a better actuation, which is favored by the fact that their oxidation and reduction potentials are close enough to occur simultaneously, as can be seen from the obtained CVs (Figure 2b), without double redox peaks nor peak separation.

Furthermore, due to the hydrophilic nature of PSS, the gel-like PEDOT fiber could absorb the aqueous pyrrole solution, allowing pyrrole monomers to diffuse into the PEDOT fiber network. Consequently, with the start of the electropolymerization process, nucleation and PPy growth could occur also in the outer part of PEDOT/PSS polymeric network, and an intermediate PEDOT/PPy layer could be formed.

Figure 3f shows a scanning electron microscope image of the typical surface morphology of the PEDOT/PPy fiber, using a 5000 s electrodeposited PPy sheath sample as an example. Macroscopic images of the fiber actuators can be found in Figure S1.

**Figure 4:**
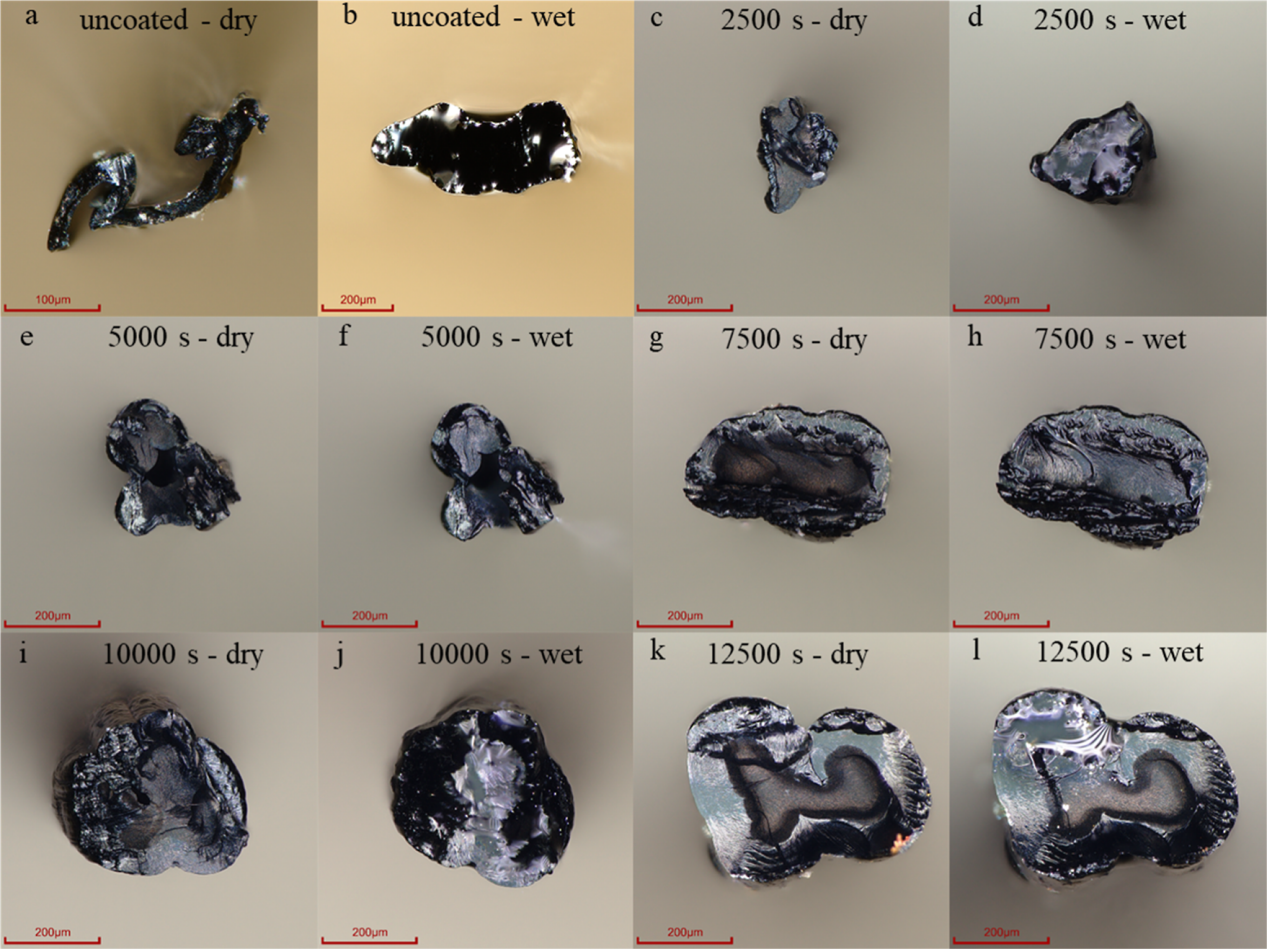
Cross-sections of uncoated fibers (a,b) and PEDOT/PPy core–sheath fibers (c–l) in dry and wet conditions with different polymerization durations as mentioned in the individual images. Note the different scale bars in (a,b) compared to (c–l).

The linear density (*T*_t_), which describes the weight of a fiber per unit length in its dry fiber state,^53^ was determined for the uncoated PEDOT and PEDOT/PPy core–sheath fibers. The cross-sectional areas of the samples were measured in wet and dry states using the previously mentioned digital light microscope. In contrast to the images presented before, the specimens were not embedded in epoxy resin to allow the fibers to soak when exposed to an aqueous environment. Example images for these examinations can be found in Figure 4.

**Figure 5:**
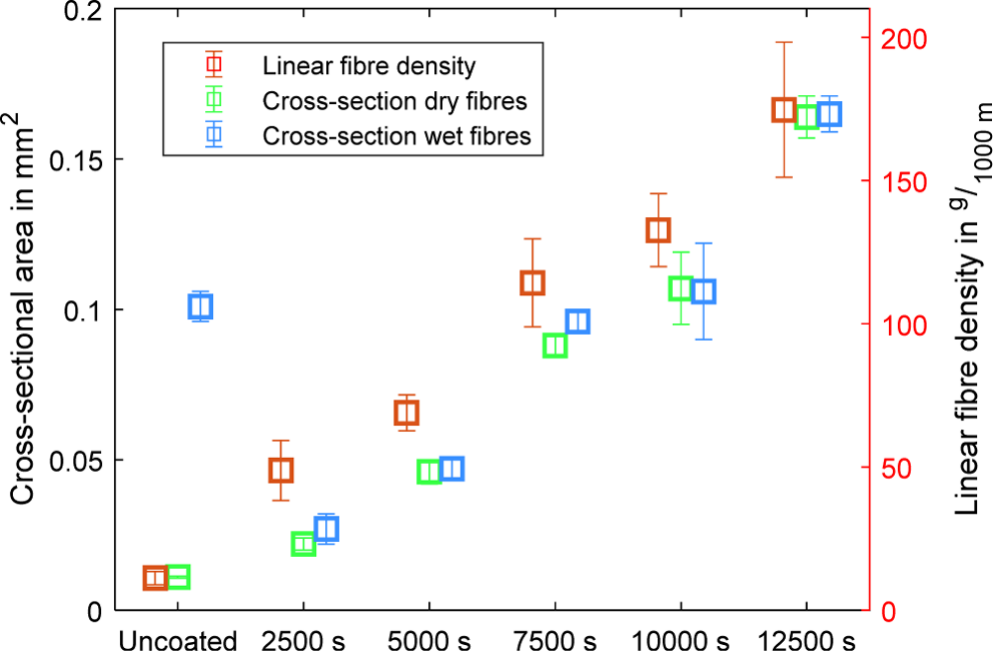
Comparison of dry and wet cross-sections and fiber count in the dry condition. Data of the uncoated fibers from ref (42). Available under a CC-BY 4.0 license © 2024 The Authors. Advanced Intelligent Systems published by Wiley-VCH GmbH.

Figure 5 presents the corresponding results. As expected, both fiber count and cross-sectional area exhibited an increase with the duration of electrodeposition. While there is a large difference in cross-section between the dry and wet states of the uncoated fibers, this difference is not present in the PEDOT/PPy core–sheath fibers. This could be due to the PPy sheath blocking the passive diffusion of water into the PEDOT core, indicating a low degree of swelling of these fibers in an aqueous environment without the influence of additional electrochemical actuation.

The 2500 and 5000 s electrodeposited samples in dry and wet states are thinner than the uncoated fiber in the wet state, although the deposition of the PPy layer is performed on the fiber in the wet state. This thinning could be caused by simultaneous electrochemically induced shrinkage of the PEDOT/PSS uncoated fiber during the PPy electrodeposition, which is an oxidative process.

**Figure 6:**
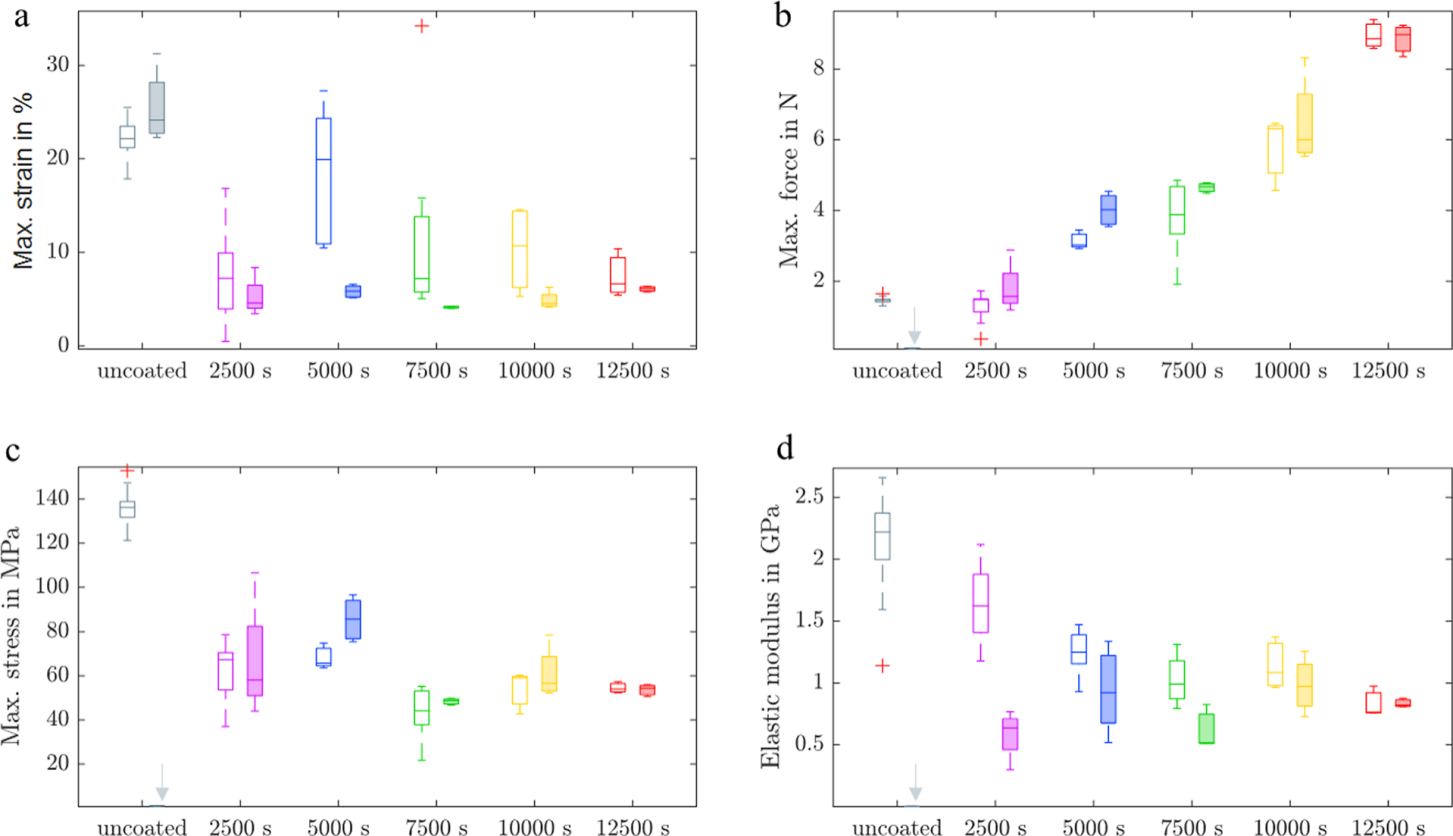
Mechanical properties of uncoated fibers and different PEDOT/PPy core–sheath fibers in dry (unfilled boxes, left-hand side) and wet (filled boxes, right-hand side) conditions. Maximum tensile strain (a), maximum tensile force (b), maximum tensile stress (c), and elastic modulus (d). Data of the uncoated fibers from ref (42). Available under a CC-BY 4.0 license © 2024 The Authors. Advanced Intelligent Systems published by Wiley-VCH GmbH.

To gain a better understanding of the fibers’ suitability for further textile processing and their strength against mechanical exposure when used as artificial muscles, mechanical properties were investigated using tensile tests in both dry and wet conditions. For the experiments in the wet condition, the fibers were soaked in deionized water for 10 min and afterward immediately clamped in the tensile testing machine. Figure 6 shows the mechanical properties of the fibers with varying electrodeposition durations in both dry (unfilled boxes) and wet (filled boxes) conditions. The maximum strains observed under both dry and wet conditions (Figure 6a) showed no clear correlation with the duration of PPy electrodeposition. In dry conditions, the PEDOT/PPy core–sheath fibers exhibited higher maximum strains, ranging from 10% to 20%, compared to the lower strains of 4% to 6% recorded in wet conditions.

A significant drawback of the uncoated fibers was their complete loss of mechanical stability upon exposure to water, characterized by a maximum force of 0.1 N, a maximum stress of 1.18 MPa, and an elastic modulus of 2.8 MPa (indicated by gray arrows in Figure 6b–d). This limitation was overcome by electrodepositing PPy, which increased the maximum stress in wet conditions by 43–75-fold. Additionally, the fiber condition had little effect on the maximum tensile forces of the individual PEDOT/PPy core–sheath fibers. Slightly higher force values were observed in the wet condition. As shown in Figure 6b, the maximum force in both conditions increased from 1.5 to 8.9 N with an increased electrodeposition duration, which is attributed to the increasing fiber cross-section. As in the maximum strain results, a clear trend regarding the maximum stress (Figure 6c) in relation to the electrodeposition duration could not be easily established, revealing no clear influence of the electrodeposition duration on the maximum tensile stress properties in dry and wet conditions. The elastic modulus (Figure 6d) of pristine PEDOT fibers was 2.2 ± 0.4 GPa in dry conditions, which was decreased with an increasing PPy sheath thickness since PPy seems to be less stiff than the PEDOT fibers. In the wet condition, the elastic modulus of pristine PEDOT fibers was dramatically decreased to 1.6 ± 1 MPa due to their high liquid uptake. The electrodeposition of a less swelling PPy sheath onto fibers leads therefore to a dramatically increased elastic modulus that is slightly enhanced with a longer electrodeposition duration.

### 2.3 Actuation Performance of the PEDOT/PPy Fiber Actuators

The linear actuation properties of straight fibers were investigated via measurements of the linear isotonic strain and isometric force. For this purpose, a 25 mm fiber actuator was mounted in an electrochemical cell containing an aqueous NaDBS electrolyte (0.1 M) at a liquid level of 20 mm. The top of the prepared fiber actuator was connected to the lever of a Dual Mode Servo System (300B, Cambridge Technology, Aurora, ON, Canada) by using glued wire hooks. Figure S2 provides a schematic representation of the measurement setup. The PEDOT/PPy core–sheath fibers were actuated using a square wave potential between −1.2 and 0.2 V vs Ag/AgCl over 10 cycles, with 150 s of reduction and oxidation duration each.

**Figure 7:**
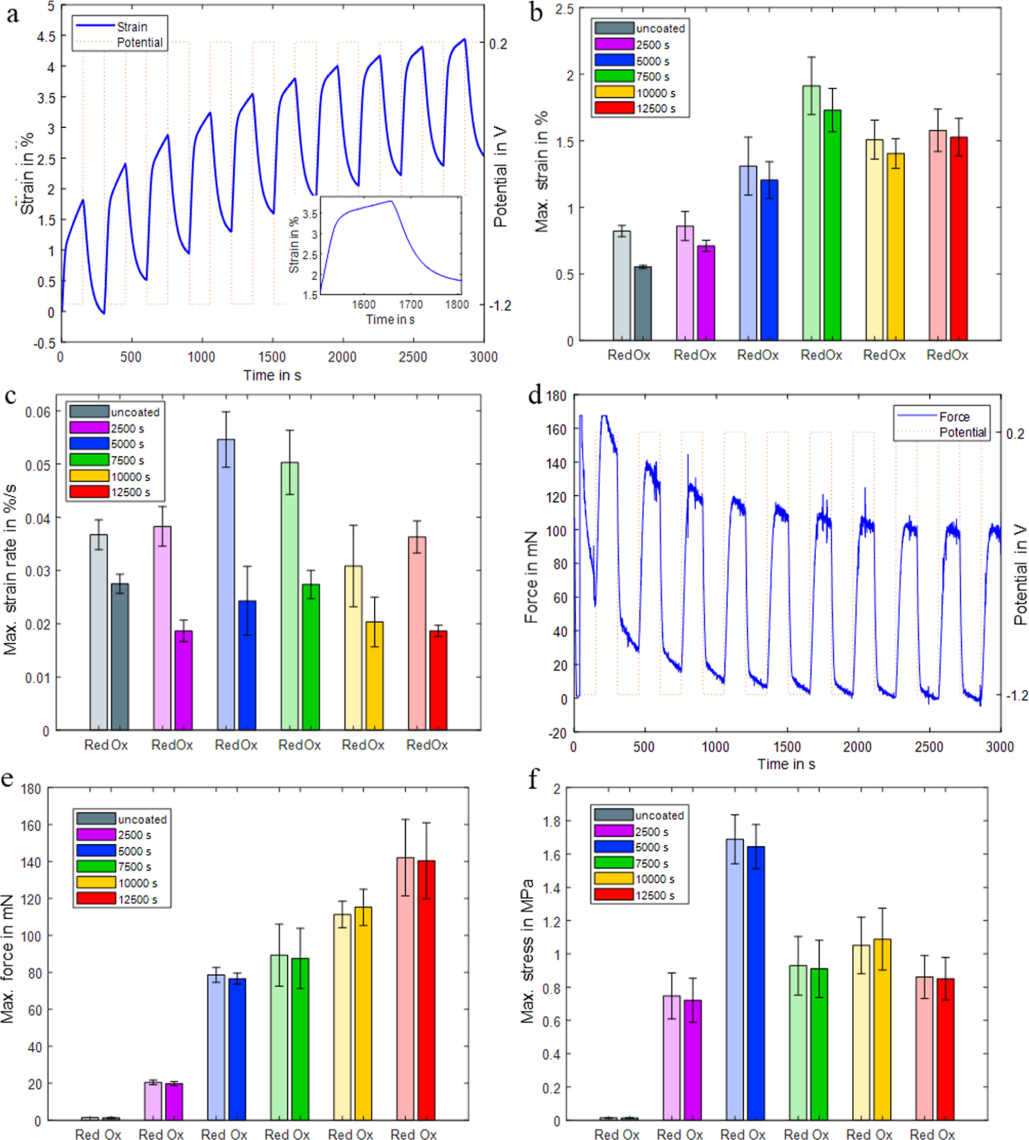
Actuation properties of the straight PEDOT/PPy fiber actuators. Example of an isotonic strain measurement (7500 s sample; inset: strain of sixth cycle) (a), calculated average max. actuation strains (b), and strain rates (c) for the different PEDOT/PPy core–sheath fiber actuators. Example of an isometric force measurement over time (7500 s sample) (d), calculated average max. actuation forces (e), and stresses (f). “Red” denotes fiber actuator reduction (leading to expansion) and “Ox” denotes fiber actuator oxidation, leading to contraction. Data of the uncoated fibers reproduced from ref (42). Available under a CC-BY 4.0 license © 2024 The Author(s). Advanced Intelligent Systems published by Wiley-VCH GmbH.

**Figure 8:**
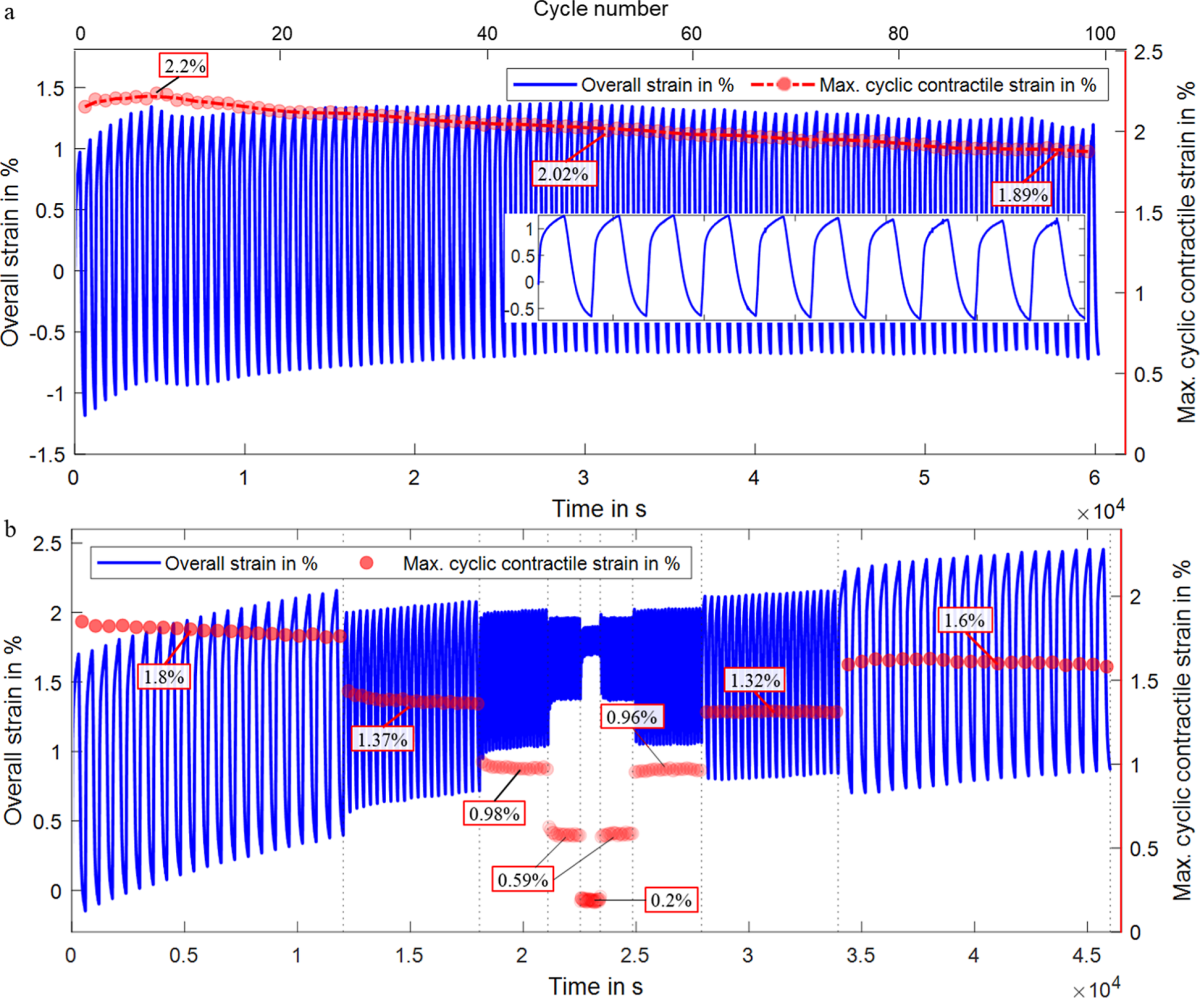
Isotonic strain measurement of a fiber actuator with 12,500 s PPy electrodeposition over 100 cycles with 300 s per oxidation and reduction (inset: close-up to last 10 cycles): (a)isotonic strain measurement of the same fiber actuator using [300, 150, 75, 35, 10, 10, 35, 75, 150, and 300] seconds for oxidation and reduction for 20 cycles each (b).

Figure 7 shows the measured actuation properties. Figure 7a shows the strain of the 7500 s PEDOT/PPy core–sheath fiber actuators as an illustration of a typical actuation of these fiber actuators. The inset showcases a close-up of the sixth cycle, illustrating the dynamics of a single actuator contraction with a reversible strain of 1.94%. Over time, it was observed that the strain initially drifted due to simultaneous initial swelling because of soaking of the fiber and mechanical creep due to the application of the pretension on the fiber. After approximately 10 cycles, the strain stabilized (see also Figure 8). To calculate the average values of the fiber actuator properties, only the last five cycles of each measurement were used.

Figure 7b shows the average maximum contraction strain during oxidation (Ox) and average maximum expansion strain during reduction (Red), indicating a significant enhancement in the actuation strain resulting from the deposition of PPy compared to that of the uncoated fibers. Regarding the PEDOT/PPy core–sheath fibers, both the maximum strain and maximum strain rate (Figure 7c) were achieved with a 7500 s PPy electrodeposition duration, resulting in an average maximum contractile strain of 1.73% at a maximum strain rate of 0.027%/s. The strain rate during reduction (fiber actuator expansion) is higher than that during oxidation (fiber contraction), which may be attributed to the actuator behaving like a spring, releasing stored energy during reduction. Another reason could be the presence of asymmetries between the oxidation and reduction reactions. The 7500 s PEDOT/PPy core–sheath fibers exhibit the highest actuation strain values, which we attribute to their elastic modulus being comparatively low (Figure 6d), facilitating their elongation and contraction during actuation.

Additionally, the PPy sheath thickness appears to reach an optimum in terms of the ion migration depth to maintain charge equilibrium throughout the radius of the fiber actuator during actuation. Actuation of CPs relies on the oxidation and reduction of the CP. These redox reactions, which are reversible, promote the migration of ions into and out of the polymer itself.^26^ This migration is influenced by the possibility that such ions move (diffuse) through the polymer matrix. A thin layer of CP thus allows a faster diffusion as the ions do not need to travel long distances through the polymer matrix to arrive at the inner part of the polymer. However, for increasing thicknesses, in the same energetic conditions, this process becomes slower, as the movement of the ions to the inner part of the polymer will take a longer diffusion time.^54^ Thus, when the time of the experiment is constant, it is possible to assume that for increasing thicknesses, it will not be possible to fully oxidize or reduce the CP, as the slow diffusion of ions would require more time. This optimal ion migration can be further confirmed by the fact that at the thickness obtained after 7500 s, the maximum reversible (redox) charge was consumed in the redox reactions (Figure 2c). As this synthesis charge is proportional to the amount of ions that could diffuse into or out of the polymer,^55^ this points again to the faradaic control of the actuation, driven by the reversible oxidation and reduction of the PEDOT/PPy core–sheath fiber with the subsequent ion migration.^55^

Figure 7d shows a typical isometric force measurement, also for a 7500 s PEDOT/PPy core–sheath fiber actuator. Due to the initial soaking-induced expansion of the fibers, they were straightened by manually lifting the lever within the first reduction (expansion) of the fibers during isometric force measurements. This resulted in a force peak in the first reduction of the fiber. Thereafter, we see a quick rise of the force in each cycle, followed by a plateau (constant force). As with the strain, the forces stabilized after five cycles. The maximum contractile force was 140 ± 20.5 mN for a 12,500 s PEDOT/PPy core–sheath fiber (Figure 7e) due to the higher cross-sectional area of PPy. Higher cross-sectional area means more material available for actuation and, thus, more force. This represents a dramatic 103-fold increase in the actuation force relative to the uncoated fibers. The maximum contractile stress of 1.64 ± 0.13 MPa was recorded for fibers with a 5000 s PPy electrodeposition duration (Figure 7f), which is a 122-fold increase in actuation stress compared to the uncoated fibers. As discussed above, a thicker PPy leads to slower diffusion of ions through the polymer chains, decreasing in fact the overall oxidation or reduction state (as could be seen in Figure 2c for the reversible redox charge), which causes a decrease in the stress produced by the fiber. Considering the fiber count, the fibers can lift more than 2000 times (2500 s) to more than 4000 times (12,500 s) their own weight. To further increase the actuation strain and reveal the PEDOT/PPy core–sheath fibers’ actuation stability over numerous cycles, we expanded the oxidation and reduction duration time to 300 s over 100 cycles using the fiber actuator with 12,500 s PPy electrodeposition duration. The straight actuator fiber showed an outstanding initial contractile linear strain of 2.2 ± 0.02% over the initial 10 cycles, 2.02 ± 0.01% over the cycles 45–55, and still 1.89 ± 0.01% after 100 cycles, which corresponds to an actuation strain decrease of 8.18% after 50 cycles and only 14.09% after 100 cycles (Figure 8a). The inset presents a detailed view of the last ten cycles, demonstrating a high reversibility. Long-term tests over 140 and 150 cycles using 10,000 and 12,500 s electrodeposition duration supported the long-term stability of these yarn actuators (Figure S3).

To assess the influence of the actuation duration, we used the same actuator fiber (12,500 s PPy electrodeposition) for another isotonic actuation strain experiment under variated oxidation and reduction durations, namely, 300, 150, 75, 35, 10, 10, 35, 75, 150, and 300 s over 20 cycles each, see Figure 8b. The recorded contractile strains were 1.8, 1.37, 0.98, 0.59, 0.2, 0.2, 0.59, 0.96, 1.32, and 1.6%. It shows again the effect of ion diffusion on the actuation. For shorter periods, we got a faster actuation, but a full oxidation/reduction was not achieved, as the ions would require more time to diffuse through the polymer matrix to the inner part of the polymer, hence a lower strain. However, the data again demonstrates a high actuation stability under different actuation durations.

## 3 Conclusions

This study demonstrates the development of novel conducting polymer actuator fibers consisting of PEDOT/PPy core–sheath fibers with a wet-spun PEDOT core and an electrodeposited PPy sheath. We experimentally investigated the influence of different PPy sheath thicknesses on the mechanical and actuation properties of these fibers. The fibers demonstrated high tensile strengths under both dry and aqueous conditions.

Regarding the electromechanical actuation, these untwisted and uncoiled linear actuator fibers demonstrated remarkable performances, achieving maximum linear isotonic contractile strains of up to 2.2% and isometric actuation forces up to 140 mN, with stresses reaching 1.64 MPa, allowing them to lift up to 4000 times their own weight.

Compared to the used and previously presented pristine PEDOT fibers,^42^ the PEDOT/PPy core–sheath fibers exhibit a dramatic more than 100-fold increase in actuation strength.

The results of the long-term experiments demonstrated that over 85% of the initial contractile strain was retained after 100 actuation cycles. This confirms the excellent stability of actuation over numerous cycles under a range of conditions, which highlights the potential for practical applications in wearables and soft robotics. Additionally, these findings establish the presented fibers as pioneering freestanding and nonfurther structurally processed CP actuator fibers, achieving the highest electrochemically induced actuation strains in such CP fibers to date. As documented, it is expected that textile processing techniques, such as coiling or knitting, further enhance the actuation properties of CP-based actuator fibers. These core–sheath fibers represent an important step toward continuous processing, which is crucial when integrating such actuators in garments.^56^ In our study, the fibers were investigated in a liquid environment, whereas in-air actuation is necessary for practical applications. To achieve this, the fibers need to be assembled into yarns, coated with ionogel, and paired to form an anode–cathode system, as we recently demonstrated.^56−58^ Such electromechanically active yarns can thereafter be woven or knitted into functional fabrics^10,32^ and garments.^13^ Thus, this work shows significant advances for the development of wearables with actuation capabilities and soft robotics driven by CPs, whether used as presented or further textile processed.

## 4 Experimental Section/Methods

### 4.1 Materials

PEDOT/PSS aqueous dispersion with a solid content between 1 and 1.3 wt %; PEDOT/PSS weight ratio 1:2.5 was purchased from Heraeus (Clevios PH 1000, Heraeus Epurio GMBH, Hanau, Germany) and stored at room temperature (RT). Dimethyl sulfoxide, acetone, isopropanol, and sulfuric acid (96%) that was also used for wet-spinning of PEDOT/PSS fibers^42^ were purchased from VWR International GMBH (Dresden, Germany) and stored under RT. Sodium dodecylbenzenesulfonate (NaDBS) and pyrrole were acquired from Merck Life Science AB (Solna, Sweden). Pyrrole was vacuum-distilled and stored at −20 °C before use. Deionized water (Milli-Q, 18.2 MΩ cm) was used for PPy syntheses and actuation experiments.

### 4.2 Polypyrrole Electrodeposition and Cyclic Voltammetry

For all electrochemical depositions of PPy onto the PEDOT fibers, we prepared 50 mm PEDOT/PSS fibers and glued a varnished metal wire hook using epoxy glue to the end of the fiber. 30 mm of the fibers was submerged into a centrifuge tube equipped with a circular positioned stainless steel mesh, which served as the counter electrode at a radial distance of ∼12 mm to the fiber. The centrifuge tube was filled with a 0.1 M pyrrole monomer in 0.1 M aqueous NaDBS solution until 25 mm of the fiber was exposed to the monomer solution. The fiber was straightened due to the weight of the hook. A MF-2052 Ag/AgCl (3 M KCl) from BASi (Bioanalytical Systems, Inc., West Lafayete, IN, USA) was inserted in the cell and served as a reference electrode, while the PEDOT fiber served as the working electrode. All electrochemical depositions and electrochemical characterizations (including actuation experiments) were assessed using an Autolab PGSTAT 204 potentiostat and Nova 2.1 software (Metrohm Ag, Herisau, Switzerland). The device was connected to the counter and reference electrodes using alligator clips and to the PEDOT fiber using a Minigrabber test lead with hook grips (Pomona Electronics Inc., Everett, WA, US). To reduce the contact resistance, the fiber was coated with silver conductive paint at the contact point.

Before the polymerization, we measured the electroactivity of the uncoated PEDOT fibers using CV between 0.2 and −1.2 V vs Ag/AgCL (3 M KCl) reference electrode at a scan rate of 10 mVs^–1^. Figure 1c shows the setups for these experiments. We used galvanostatic electropolymerization technique with a set constant current of 0.5 mA over variated polymerization times to realize different PPy fiber sheath thicknesses, namely, 2500, 5000, 7500, 10,000, and 12,500 s. After the electrodepositions, we repeated the CV measurements using the same specifications as described above. All electrodepositions and CV were performed under RT.

### 4.3 Fiber Characterization

For investigation of the fiber count or linear density, samples of 25 mm length from all PEDOT/PPy fibers in the dry state were weighted using an Entris II Microbalance (Satoris Lab Instruments GmbH & CO. KG, Göttingen, Germany).

The microscopic cross-sectional images in Figure 3 were captured by using a digital microscope (Dsx 1000 by Olympus, Shinjuku, Japan). Here, specimens were prepared by embedding the fibers vertically in epoxy resin under vacuum. To ensure a smooth surface of the specimens, the cured specimens were processed with a grinding and polishing device (TefraPol-15, Struers A/S, Denmark) up to a diamond suspension of 1 μm. The SEM image in Figure 3f was captured by using an ESEM QUANTA FEG 250 scanning electron microscope (FEI Company, Hillsboro, OR, USA).

The cross-sectional area was determined using the previously mentioned digital microscope and the area measurement tool of ImageJ (1.54d) software (Figure 4). For dry and wet fiber states, we cut the fibers into five pieces of equal length and glued the fibers to the edge of a microscope slide using double-sided adhesive tape. Afterward, we again cut the fibers ca. 1 mm above the glued area so that the viewing axis was perpendicular to the cutting plane of the free-standing fiber tips. The cross-sections in the dry state were captured in this setup. We immersed the fibers directly after capturing the cross-sectional images in the dry state for 10 min in deionized water before capturing the images to determine the cross-section in liquid. It should be noted that no additional electrical potential was applied during these measurements.

The tensile properties of the fibers (Figure 6) were examined according to DIN EN ISO 5079:2021-02 using a using a zwickiLine Z2.5 tensile testing machine (ZwickRoell GmbH & Co KG, Germany) with a 50 N load cell. All tests were repeated 5–10 times. For evaluating the tensile properties of the fibers in an aqueous environment, each fiber was immersed in deionized water for 10 min prior to the tensile test.

### 4.4 Actuation Experiments

The electrochemical cell was constructed using an inverted centrifuge tube with an open end, measuring 25 mm in height. The fiber actuators were threaded through an aperture in the lid of the cover and affixed with an epoxy resin adhesive. The electrochemical cell was filled with a 0.1 M aqueous NaDBS electrolyte until a length of 20 mm from the fiber actuators was exposed to the electrolyte. For all actuation experiments, we used the same counter and reference electrodes and the same potentiostat as mentioned above. Every cyclic actuation experiment was repeated three times. We exclusively considered redox cycles 6–10 for the calculation of all given actuation properties; that is, redox cycles 1–5 were not considered in the calculations. For isometric force measurements, we straightened the fibers during the start of the first reduction by manually adjusting the lever until a tensile force of ∼160 mN was reached and kept the lever in this position during the entire following measurement.

To calculate the isometric actuation forces, we calculated the tensile force during oxidation and the force relief during reduction of the fiber. The tensile force during oxidation was calculated by subtracting the average minimum force during the preceding reduction cycle from the average maximum force peak during the corresponding oxidation. The force relief during a reduction was calculated by subtracting the average minimum force while subtracting the corresponding reduction from the average maximum force during the preceding oxidation cycle.

To calculate the maximum tensile stresses, actuation forces were divided by the respective fiber cross-sections in a liquid environment. Note that these cross-sections were recorded in a fabricated neutral state (no applied potential) as described above, while oxidation decreases the fiber cross-section. The specified stresses must therefore be interpreted as minimum actuation stresses since the cross-section shrinks while oxidation.^59^

A preforce of ∼12.5 mN was set up at the lever arm hardware prior to the isotonic strain experiments and remained constant throughout the entire measurement, i.e., all reported isotonic strain measurements were performed at a constant tensile force of 12.5 mN. The expansion strain during reduction was calculated by subtracting the maximum strain during reduction from the minimum strain observed during the previous oxidation. Similarly, the contractile strain during oxidation was calculated by subtracting the maximum strain observed during reduction from the minimum strain observed during subsequent oxidation. This calculation was also used to calculate the maximum contractile strains (red dotted lines) in the long term and variation of actuation times experiments. The strain rate in Figure 7d was calculated by determining the maximum values of the first derivative of the recorded strains during the last five oxidation cycles.

## Supporting Information Available

Examples of the PEDOT/PPy core–sheath actuator fibers, schematic image of the actuation experiments, and additional long-term actuation experiments (PDF)
