# Supplementary material for: PEDOT/Polypyrrole Core–Sheath Fibers for Use as Conducting Polymer Artificial Muscles
Source: ACS Appl Mater Interfaces. 2025 Jan 16;17(4):6901–12. doi: 10.1021/acsami.4c17667 (PMC11788997; doi:10.1021/acsami.4c17667)
Supplement: Supplementary file 1 — am4c17667_si_001.pdf [file am4c17667_si_001.pdf]

Supporting Information

PEDOT/Polypyrrole Core-Sheath Fibers for Use as Conducting Polymer Artificial Muscles

Mathis Bruns[1], Shayan Mehraeen[2], Jose G. Martinez,[2] Chokri Cherif[1], and Edwin W.H. Jager[2*]*

[1] Institute of Textile Machinery and High Performance Material Technology (ITM)

TUD Dresden University of Technology

01062 Dresden, Germany

[2] S. Mehraeen, J. G. Martinez, E. W. H. Jager

Sensor and Actuator Systems

Department of Physics, Chemistry and Biology (IFM)

Linköping University

Linköping SE-581 83, Sweden

Email: jose.gabriel.martinez@liu.se, edwin.jager@liu.se

Figure S1 presents a macroscopic view on the prepared PEDOT/PPy core-sheath fiber actuators, with 2500 s, 5000 s, 7500 s, 10 000 s, 12 500 s PPy electrodeposition duration samples from left to right. Each fiber actuator has a wire glued to the lower end to mount the actuator to the actuation test device. The lower 25 mm of every fiber actuator represents the PEDOT/PPy core-sheath fiber, while the fiber sections above are uncoated. The uncoated sections including contact areas with silver conductive paint to reduce the transition resistance while electrodeposition and actuation experiments.

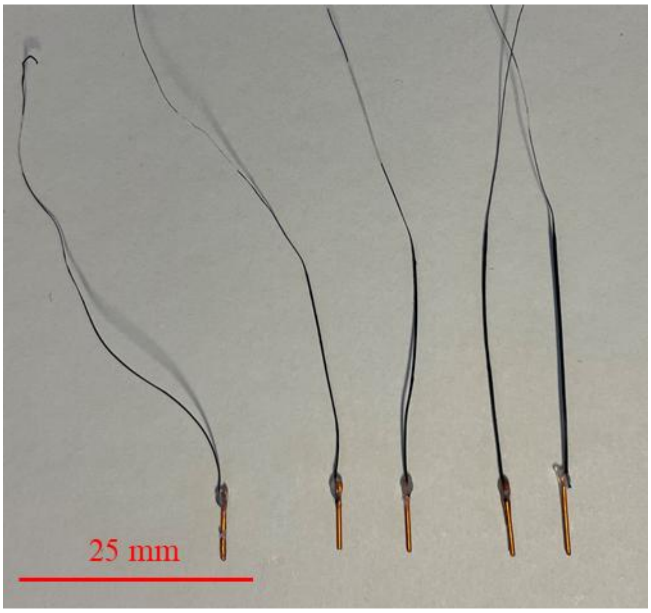

Figure S1. Image of the produced PEDOT/PPy core-sheath fiber actuators. From left to right: 2500 s, 5000 s, 7500 s, 10000 s, 12500 s PPy electrodeposition duration.

Figure S2 images a schematic view of the used actuation testing setup. The different PEDOT/PPy core-sheath fiber actuators with the previous attached vanished metal hook were threaded through the cap of the centrifugal tube and fixated using epoxy resin adhesive and positioned under the lever arm. A stainless steel mesh was used as the counter electrode while an Ag/AgCl reference electrode was used as a reference electrode. The fiber actuators hook was connected to the hook of the lever and 12.5 mN preforce was set up for all isotonic strain measurements or the lever was lifted. For isometric force measurements, the fibers were

straightened during the start of the first reduction by manually adjusting the lever until a tensile force of ~160 mN was reached and kept the lever in this position during the entire following measurement.

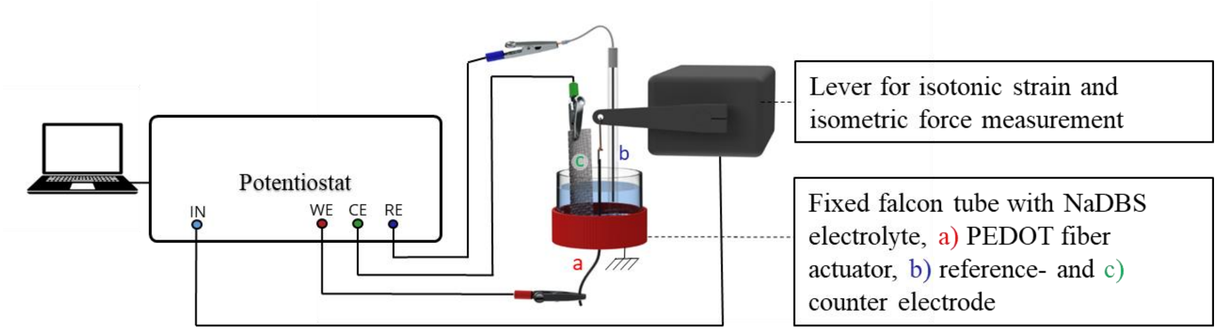

Figure S2. Schematic image of the used setup for all actuation experiments described. Image reproduced from our previous work.[1]

In addition to the experiments presented in Error! Reference source not found., actuation stability over numerous cycles were evaluated using the example of an actuator fiber with a 10 000 s electrodeposited PPy sheath.

The sample was prepared and evaluated using the same setup for the measurements in Error! Reference source not found.a (150 seconds for every oxidation and reduction) but the test was conducted over 140 cycles. **Figure S3**a shows the measured actuation strain. The fiber actuator showed a high-level of actuation strain and -stability with an initial strain of 1.35 ± 0.05% over the initial 10 cycles; 1.14 ± 0.01% over cycles 65 - 75 and 1.16 ± 0.01% over the last 10 cycles which corresponds to an actuation strain decrease of only 15.6% after 70 cycles and only 14% after 150 cycles.

Similar experiments were repeated using a 12 500 s electrodeposited PEDOT/PPy core-sheath fiber actuator. The actuator was tested first using again 150 s for oxidation and reduction over 150 cycles. The initial contractile linear strain was 1.63 ± 0.09% over the initial 10 cycles, 1.58 ± 0.01 % over cycles 70 – 80 and still 1.51 ± 0.01% over the last ten cycles which corresponds

to an actuation strain decrease of only 3% after 75 cycles and 7.4% after 150 cycles (**Figure S3**b).

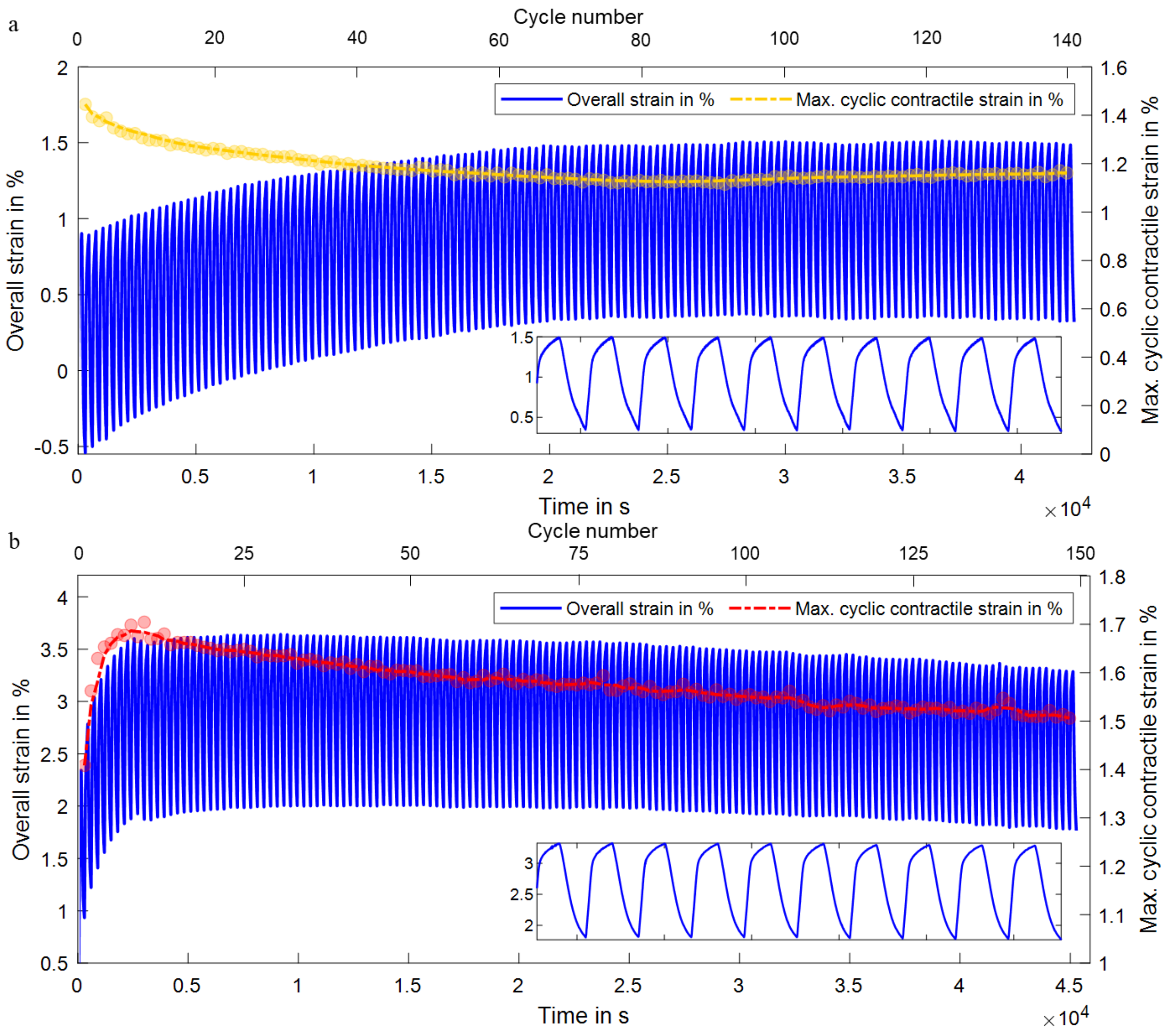

Figure S3. Long-term isotonic actuation strain experiments. PEDOT/PPy core-sheath fiber actuator with a) 10000 s PPy electrodeposition with 150 s per actuation over 140 cycles; b) 12500 s PPy electrodeposition with 150 s per actuation over 150 cycles. Insets: close-up on the last ten cycles.

References

(1) Bruns, M.; Mehraeen, S.; Martinez, J. G.; Mersch, J.; Kruppke, I.; Jager, E.; Cherif. C. A straightforward Approach of Wet-Spinning PEDOT: PSS Fibers for Use in all Conducting Polymer-Based Textile Actuators. *Advanced Intelligent Systems* **2024**, *2400315* (2400315), 2400315.
